# Consanguineous Marriages

**Published:** 1867-10

**Authors:** Robert Newman

**Affiliations:** 118 W. Houston St., New York


### *Consanguineous Marriages.*

*118 W. Houston St., New York. July, 1867.*

SIR:—At the late meeting of the "Medical Society of the State of New York," it was resolved:—"That a Committee be appointed to investigate and report upon the result of consanguineous marriages, etc."

If such marriages come under your observation, you will confer a favor by answering the following questions, and transmitting such report, before November next, to the undersigned, one of the Committee appointed:—

1. Name (initials) and age of HUSBAND.
2. Nativity.
3. Age when married.
4. Constitution.
5. Health, deformities, peculiar diathesis.
6. Health of his family, hereditary diseases, deformities, etc.
7. Name (initials) of WIFE.
8. Nativity.
9. Age when married.
10. Constitution.
11. Health, deformities, peculiar diathesis.
12. Health of her family, hereditary diseases, deformities, etc.
13. How are the parties related to each other?
14. How long married?

15. How many children, or sterility?
16. Abortions; cause; how many, and at what period?
17. Children died, at what age, and from what diseases?
18. The constitution, age, and present health of living children, deformities, mental conditions, idiocy, cretinism, deaf, mute, blind, epilepsy, albinism, insane, etc.
19. Remarks and other information.

Hoping to receive your valuable coöperation for the advancement of medical science,

I remain, yours, most respectfully,
ROBERT NEWMAN, M.D.

